# Unintended pregnancy and gender inequality worldwide: an ecological analysis

**DOI:** 10.1136/bmjgh-2024-016573

**Published:** 2025-03-31

**Authors:** Gilda Sedgh, Jonathan Marc Bearak

**Affiliations:** 1 Guttmacher Institute, New York, New York, USA

**Keywords:** Maternal health, Global Health, Epidemiology

## Abstract

Unintended pregnancy compromises many women’s and girls’ ability to pursue the lives that they want. The conditional unintended pregnancy rate (CUPR) is a measure of unintended pregnancy among women who wish to avoid getting pregnant. Using the CUPR, we explore the relationship between gender inequality and unintended pregnancy across 132 countries. We used gender inequality indicators from the UNDP Human Development Report and estimates of the incidence of unintended pregnancy published by the Guttmacher Institute and WHO. We regressed the CUPR on several measures of gender inequality using least squares with a percentile bootstrap to account for sampling error and the additional uncertainty in the model-based unintended pregnancy estimates. We find that unintended pregnancy is positively correlated with multiple composite measures of gender inequality, even after controlling for countries’ levels of economic development. Of the components of gender inequality, gender disparities in educational attainment were most strongly correlated with unintended pregnancy in multivariable regressions. We also find that female educational attainment is a stronger predictor of the CUPR than male educational attainment. Analyses with the standard unintended pregnancy rate, a measure that does not take into account differences across settings in the proportion of women who wish to avoid getting pregnant, obscured the strength of the observed relationships. Further exploration of the factors underlying this relationship can inform policies to improve the quality of women’s lives.

**Summary box**

- Tracking unintended pregnancy and its correlates can inform policies and programs aimed at reducing its incidence.
- The conditional unintended pregnancy rate (CUPR) measures the incidence of unintended pregnancy among women who wish to avoid getting pregnant, rather than among all women.
- Gender inequality is positively correlated with the CUPR, even after holding economic development constant. Among various components of gender equality, gender disparities in education show the strongest relationship with unintended pregnancy rates.
- The strengths of these associations are substantially attenuated when the CUPR is replaced with the standard unintended pregnancy rate (UPR), because the UPR does not account for variations in the proportion of women who wish to avoid pregnancy across settings.
- Understanding these relationships can help shape policies aimed at reducing gender inequality and improving reproductive health outcomes for women worldwide.

## Introduction

 Unintended pregnancy compromises many women’s and girls’ ability to pursue an education, participate in social and economic activities, and pursue the lives that they want.^1^,^4^ It also carries steep costs to healthcare systems.^5^ At the aggregate level, unintended pregnancy hinders social and economic development, the theme of the United Nations Sustainable Development Goals.^2^ Societal pressures to become mothers, reproductive coercion, shortfalls in the quality and reach of family planning programmes, and other barriers to accessing and using sexual and reproductive health services contribute to women’s inability to exercise their right to determine whether and when to bear children. Tracking global trends in unintended pregnancy helps monitor progress in overcoming these obstacles.

The 2022 State of the World Population Report published by the United Nations (UN) Population Fund included cross-sectional analyses demonstrating that gender inequality, represented by the UN’s Gender Inequality Index (GII),^6^ is associated with unintended pregnancy.^2^ To more extensively test and better understand this relationship, we build on that analysis by using a refined measure of unintended pregnancy, additional measures of gender inequality and estimates of both indicators from multiple time periods over a 30-year period. We also control for potential confounders of the relationship between unintended pregnancy and gender inequality, within the constraints of an ecological analysis. We first discuss the refined measure of unintended pregnancy and its advantages for elucidating disparities in the incidence of unintended pregnancy across populations. We then report on our analyses of the relationship of gender inequality with unintended pregnancy and its outcomes, and we conclude with a discussion of the policy implications of our findings.

## Refined measure of unintended pregnancy

In nationally representative surveys of women, births are typically classified as unplanned if they were reported by women to have been unwanted or mistimed at the time of conception. Unintended pregnancies are comprised of unplanned births, abortions and miscarriages of pregnancies that were unintended. Until now, the unintended pregnancy rate (UPR) has been commonly used to monitor the incidence of unintended pregnancy.^7^,^15^ This rate is the number of unintended pregnancies for every 1000 women of reproductive age (15–49 years old). Model-based estimates of the incidence of unintended pregnancy at the global, regional and country levels have been made by the Guttmacher Institute and the WHO for 5-year periods from 1990 to 2019.^10 11 16^

The most recent estimates of unintended pregnancy convey that the 121 million unintended pregnancies that occurred annually worldwide in 2015–2019 correspond to a rate of 64 per 1000 women aged 15–49 years.^10 11^ They also convey that the incidence of unintended pregnancy varies widely across regions, ranging from 39 per 1000 women of reproductive age in high-income European countries (hereafter referred to as ‘HIC-Europe’) to 91 in Sub-Saharan Africa.

However, only women who wish to avoid pregnancy are at risk of having an unintended pregnancy, and the UPR does not take into account an important fact: the proportion of women who wish to avoid pregnancy can vary significantly across populations.^13 17^ In effect, the rate assumes that all women are at risk of having an unintended pregnancy, and this is not the case.

Demographers typically define women at risk of unintended pregnancy as those who have said in surveys that they are married or sexually active and either are using contraception or have stated that they do not want to have a child for at least 2 years. In the past decade, the UN began periodically estimating the number of women at risk of unintended pregnancy worldwide and for every region and country of the world.^18^,^20^

Researchers recently used these UN estimates of the number of women at risk of unintended pregnancy and the Guttmacher-WHO estimates of the number of unintended pregnancies to develop a refined measure of unintended pregnancy, the conditional unintended pregnancy rate (CUPR).^13^ The CUPR is the number of unintended pregnancies per 1000 sexually active reproductive-age women *who wish to avoid pregnancy*, that is, per 1000 women at risk of an unintended pregnancy.^13^

**Figure 1:**
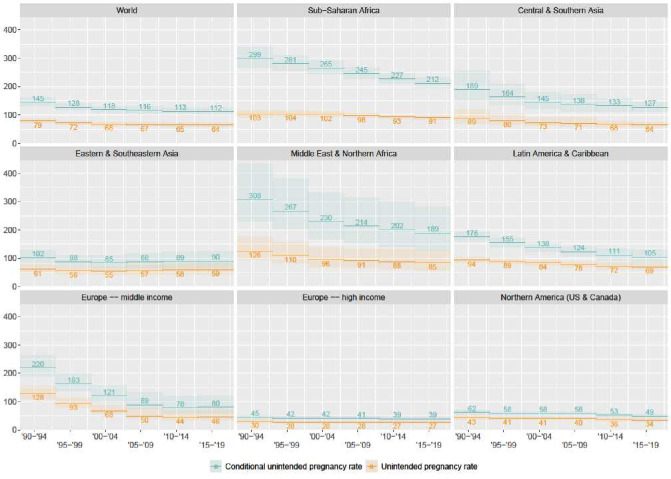
Trends in unintended pregnancy by region.

More than two-thirds of women (70%) in HIC-Europe want to avoid pregnancy, whereas only 43% of women want to avoid pregnancy in Sub-Saharan Africa.^19^ Thus, as reported previously,^13^ while the UPR in Sub-Saharan Africa is about three times the rate in HIC-Europe, the CUPR is more than five times higher in Sub-Saharan Africa than in HIC-Europe, at 207 and 39, respectively (figure 1). This means that roughly one out of every five women who wish to avoid getting pregnant in Sub-Saharan Africa nevertheless get pregnant every year, compared with one in twenty-five in HIC-Europe.

The CUPR also reveals important trends that are not apparent with the UPR. For example, the UPR declined by just 12% in Sub-Saharan Africa over the 30 years for which estimates are available, whereas the CUPR declined by 30% over the same period.^13^ This suggests that family planning programmes have had a bigger impact in the African subcontinent than the standard measure indicates. The CUPR and the UPR show similar declines in Europe and Northern America.

In sum, regional differences and trends in the CUPR convey two important points: first, the inequities between countries in the incidence of unintended pregnancy have been much larger than the UPR would indicate. Second, these inequities have grown smaller—a finding not reflected in the UPR.

To explore whether and how gender inequities are associated with the incidence of unintended pregnancy among women at risk of getting pregnant, we examined the relationship between gender inequality and unintended pregnancy using model-based estimates of the UPR and CUPR previously published and indicators of gender inequality from the United Nations Development Programme’s (UNDP) Human Development Report.^21^

The primary outcome of interest is unintended pregnancy because we posit that gender inequality can affect whether women who want to avoid pregnancy are able to take the necessary measures to do so. However, we also separately examined the association between gender inequality and the conditional unplanned birth rate (CUBR) (the number of unplanned births per 1000 women wishing to avoid pregnancy), which additionally captures whether women are able to avoid carrying unplanned pregnancies to term. As a complement to this analysis, we examine the relationship between gender inequality and the proportion of unintended pregnancies aborted. Hence, we posit that gender inequality is directly correlated with unintended pregnancy and unplanned birth rates and, because gender inequality can limit autonomy and access to care, inversely correlated with the proportion of unintended pregnancies that end in abortion.

## Data and analysis

Country-specific estimates of unintended pregnancy, unplanned births and abortion have been published for 150 countries, and of these, the variables in our analysis were available for 132 (online supplemental appendix table 1).

The UNDP has developed two composite indices that could be used to represent gender inequality. The GII is a composite measure that reflects gender-based inequalities in economic activity, educational and political empowerment, and reproductive health.^6^ Economic activity is based on female and male labour force participation rates. Empowerment is measured by the percentage of parliamentary seats held by women and gender differences in obtaining at least a secondary school education. The health dimension is represented by the maternal mortality ratio (MMR) and the adolescent fertility rate. Higher GII scores correspond with higher levels of gender inequality.

The Gender Development Index (GDI) is comprised of measures of gender differentials in health outcomes and access to healthcare, calculated from gender differences in life expectancies at birth; education, measured by expected years of schooling at childhood as well as mean years of schooling for adults ages 25 years and older; and command over economic resources, measured by female and male estimated gross earned income per capita.^6^ Unlike GII, higher GDI scores correspond with higher levels of gender *equality*.

We conducted bivariate analyses using the GII and GDI (the composite variables) as predictors of the CUPR in 2015–2019. Because the GII includes health measures directly related to pregnancy, namely the MMR and the adolescent birth rate (ABR), we also conducted analyses in which we removed these two factors from the GII. We additionally ran bivariate models with the component variables of these composite indicators, except for the MMR and ABR, as predictors of the CUPR. We then ran models testing these associations, controlling for overall economic development, represented by countries’ gross domestic product per capita (GDPPC). To examine which among the component variables was most strongly associated with unintended pregnancy, we also estimated multivariable models that controlled for all of them and GDPPC simultaneously. We repeated these models, replacing unintended pregnancy with unplanned births and the proportion of unintended pregnancies aborted.

We conducted an additional analysis that examined whether associations remained significant if we replaced each of the gender ratio variables with their numerators and denominators; for example, we replaced the gender ratio in expected years of schooling with variables for female expected years of schooling and male expected years of schooling. In these analyses, the male-specific variables serve as proxies for overall progress on these aspects of social and economic development.

As an additional check, we estimated regressions replacing GII and GDI with the Global Gender Gap Index (GGI), a metric developed by the World Economic Forum to assess gaps between women and men in participation in the workforce, educational attainment, health outcomes and political empowerment.^22^ We did not conduct analyses with the components of the GGI (gender gaps in economic opportunities, education, health and political leadership) because they were conceptually very similar to the components of the GII and GDI and because data representing these components were not readily available. Higher GGI scores correspond with higher levels of gender equality.

As an additional test of the robustness of our findings, and to ensure that they are not driven by patterns in any one region, we performed a regional jackknife. In this procedure, separately for each region, we deleted all the data from that region and re-estimated each of the regression models.

Finally, we re-estimated our regressions for each 5-year period for which data were available (with fewer countries included in earlier years due to lack of available data in the Human Development Report).

We estimated the regression coefficients using least squares. We produced 95% CIs using a percentile bootstrap with 10 000 iterations. In each iteration of the bootstrap, we randomly sampled countries. We then replaced the CUPRs for those countries with those from a randomly sampled iteration from the Markov chains produced by the previously published unintended pregnancy model, to account for uncertainty in the unintended pregnancy estimates. We report point estimates from a regression that used each country’s mean CUPRs and CIs from the 2.5th and 97.5th percentiles of the bootstrap distribution.

We standardised the composite variables and used the natural logs of the component measures, the CUPRs and GDP per capita. As such, exponentiating the coefficients for the composite variables gives the percentage change in the CUPR for every SD change in the composite variable, and the coefficients for the component measures represent the percentage change in the CUPR corresponding with every 1% change in the component.

This study used secondary data that were previously collected and are publicly available. These data are national level estimates and as such they do not include information on individuals.

## Correlations between unintended pregnancy and gender equality

The CUPR was associated with the GII, the GDI and the GGI in bivariate analyses (table 1). These relationships held after controlling for GDP per capita, with coefficients of 0.59, –0.37 and −0.30, respectively. In other words, for example, a 1 SD improvement in the GII is associated with a 45% $\left(1\;-\;e^{-0.59}\approx 0.45\right)$ decline in the CUPR. The relationship between GII and the CUPR is attenuated, but still strong, after we remove the health component from the GII indicator.

**Table 1:** Relationships between gender inequality indicators and their components with unintended pregnancy and its outcomes in 2015–2019

		Relationship with CUPR (95% CI)		Relationship with UPR (95% CI)		Relationship with CUBR (95% CI)		Relationship with PUA (95% CI)	
Source	Variable*	Univariableassociations	Controlling for GDPPC	Univariable associations	Controlling for GDPPC	Univariable associations	Controlling for GDPPC	Univariable associations	Controlling for GDPPC
	Gender Inequality Index (with health)	0.59 (0.51, 0.66)	0.55 (0.45, 0.64)	0.44 (0.35, 0.52)	0.44 (0.34, 0.55)	0.64 (0.54, 0.77)	0.62 (0.48, 0.78)	−0.08 (−0.28, 0.07)	−0.10 (−0.31, 0.08)
		p=0.00	p=0.00	p=0.00	p=0.00	p=0.00	p=0.00	p=0.29	p=0.28
	Gender Inequality Index (without health)†	0.28 (0.15, 0.40)	0.20 (0.08, 0.30)	0.14 (0.03, 0.25)	0.09 (−0.02, 0.18)	0.19 (0.01, 0.36)	0.11 (−0.06, 0.26)	0.12 (−0.08, 0.30)	0.13 (−0.06, 0.31)
		p=0.00	p=0.00	p=0.01	p=0.11	p=0.03	p=0.19	p=0.22	p=0.16
	Gender Development Index	−0.37 (−0.48, −0.28)	−0.31 (−0.40, −0.22)	−0.20 (−0.30, −0.12)	−0.17 (−0.27, −0.08)	−0.42 (−0.56, −0.30)	−0.36 (−0.50, −0.25)	0.07 (−0.09, 0.27)	0.08 (−0.09, 0.29)
		p=0.00	p=0.00	p=0.00	p=0.00	p=0.00	p=0.00	p=0.37	p=0.35
	Global Gender Gap Index	−0.30 (−0.42, −0.18)	−0.23 (−0.34, −0.12)	−0.14 (−0.24, −0.05)	−0.10 (−0.20, −0.01)	−0.17 (−0.31, 0.00)	−0.09 (−0.24, 0.07)	−0.19 (−0.36, −0.01)	−0.20 (−0.38, −0.03)
		p=0.00	p=0.00	p=0.00	p=0.03	p=0.05	p=0.26	p=0.04	p=0.03
GII	ln F/M secondary education	−1.27 (−1.68, −0.96)	−0.98 (−1.37, −0.67)	−0.72 (−1.10, −0.42)	−0.54 (−0.93, −0.24)	−1.65 (−2.13, −1.32)	−1.40 (−1.90, −1.04)	0.58 (−0.01, 1.27)	0.62 (−0.01, 1.32)
		p=0.00	p=0.00	p=0.00	p=0.00	p=0.00	p=0.00	p=0.05	p=0.05
GDI	ln F/M expected years of schooling	−2.88 (−4.01, −1.94)	−2.46 (−3.70, −1.51)	−1.44 (−2.58, −0.55)	−1.31 (−2.54, −0.39)	−3.49 (−4.84, −2.42)	−3.23 (−4.65, −2.16)	0.99 (−0.59, 2.82)	1.21 (−0.53, 3.19)
		p=0.00	p=0.00	p=0.00	p=0.00	p=0.00	p=0.00	p=0.22	p=0.18
GDI	ln F/M mean years of schooling	−1.45 (−1.90, −1.10)	−1.09 (−1.53, −0.74)	−0.81 (−1.25, −0.46)	−0.59 (−1.04, −0.24)	−1.79 (−2.32, −1.39)	−1.48 (−2.02, −1.03)	0.50 (−0.20, 1.27)	0.53 (−0.21, 1.32)
		p=0.00	p=0.00	p=0.00	p=0.00	p=0.00	p=0.00	p=0.15	p=0.15
GII	ln F/M labour force participation	−0.08 (−0.54, 0.52)	−0.18 (−0.58, 0.49)	0.06 (−0.31, 0.54)	0.02 (−0.32, 0.59)	0.49 (−0.20, 1.33)	0.41 (−0.19, 1.31)	−0.79 (−1.54, 0.04)	−0.82 (−1.58, 0.00)
		p=0.83	p=0.60	p=0.72	p=0.84	p=0.16	p=0.18	p=0.06	p=0.05
GII	ln percentage of parliamentary seats held by women	−0.24 (−0.42, −0.06)	−0.12 (−0.28, 0.04)	−0.12 (−0.27, 0.04)	−0.04 (−0.18, 0.11)	−0.07 (−0.30, 0.17)	0.05 (−0.16, 0.29)	−0.24 (−0.49, 0.00)	−0.26 (−0.51, −0.03)
		p=0.01	p=0.16	p=0.16	p=0.64	p=0.58	p=0.59	p=0.05	p=0.03
GDI	ln F/M gross national income (per capita)	−0.23 (−0.62, 0.28)	−0.38 (−0.71, 0.13)	−0.07 (−0.40, 0.34)	−0.15 (−0.45, 0.29)	0.36 (−0.23, 1.16)	0.22 (−0.29, 1.04)	−0.77 (−1.48, −0.13)	−0.78 (−1.53, −0.13)
		p=0.35	p=0.12	p=0.74	p=0.48	p=0.23	p=0.43	p=0.02	p=0.02
GDI	ln F/M life expectancy	−2.72 (−7.34, 2.02)	−3.77 (−8.08, 0.51)	−0.64 (−4.68, 3.43)	−1.40 (−5.30, 2.48)	−7.84 (−14.04, −2.04)	−9.15 (−15.23, −3.73)	5.73 (−0.85, 12.27)	6.12 (−0.49, 12.90)
		p=0.24	p=0.08	p=0.75	p=0.44	p=0.01	p=0.00	p=0.08	p=0.07

Higher GII scores correspond with higher levels of gender inequality. Higher GDI and GGI scores correspond with higher levels of gender equality.

* We standardised the index variables (GII, GDI and GGI) and took the natural log of the others (the rates, GDPPC and the variables representing components of the index variables).

† To create the variable representing GII without the reproductive health component, we multiplied the two remaining components and took the square root of the result.

CUBRconditional unplanned birth rateCUPRconditional unintended pregnancy rateGDIGender Development IndexGDPPCgross domestic product per capitaGGIGlobal Gender Gap IndexGIIGender Inequality IndexPUApercent of unintended pregnancies ending in abortionUPRunintended pregnancy rate

In bivariate analyses with each of the components of GII and GDI, the indicators of education and political empowerment—the gender ratio in expected years of schooling, mean years of schooling, the percentage who have had at least a secondary school education and the percentage of parliamentary seats held by women—were all strongly associated with the CUPR. The three indicators of disparities in educational attainment remained significantly associated with the CUPR after controlling for overall GDP per capita. In addition, the coefficient for the gender ratio in life expectancy became marginally significant. According to these results, a 10% improvement in the ratio of females to males having at least a secondary education was associated with a 9.8% decrease in the CUPR, holding GDP per capita constant

All three measures of disparities in educational attainment remained strongly correlated with CUPR in multivariable analyses, controlling for the other key components of gender inequality from the GII and GDI (gender ratios in gross national income, labour force participation and life expectancy at birth) and GDPPC (online supplemental appendix table 4). These indicators of educational attainment were even more strongly correlated with the CUBR than with the CUPR. The gender ratio in life expectancy was also strongly and significantly correlated with the CUBR. For education and life expectancy, the larger associations with CUBR compared to CUPR corresponded with the finding that improvements in these gender ratios were associated with larger proportions of unintended pregnancies ending in abortion; for education, whether this was significant depended on the metric.

When we estimated regressions that replaced the gender ratios with separate variables for their numerators and denominators (separate variables for males and females), the female-specific indicators were generally more strongly associated with the CUPR than male-specific indicators. More specifically, associations between CUPR and women’s expected years of schooling, the percentage who achieved some level of secondary education, life expectancy and gross national income per capita (GNIPC), controlling for GDPPC, were all strongly to marginally significant, while the corresponding male predictors had very weak associations (table 2). The only significant association with a male predictor was male labour force participation and this was opposite-signed: in countries with higher male labour force participation, net of female labour force participation and GDPPC, there were more unintended pregnancies per 1000 women wanting to avoid pregnancy.

**Table 2:** Relationships with the natural log of the conditional unintended pregnancy rate in 2015–2019 in models with female and male components of gender ratios and gross domestic product per capita

Source	Variable	Female	Male
GII	ln secondary education	−0.36 (−0.80, 0.04)	−0.14 (−0.75, 0.48)
		p=0.079	p=0.650
GDI	ln expected years of schooling	−1.03 (−2.19, 0.00)	−0.59 (−1.94, 0.78)
		p=0.050	p=0.387
GDI	ln mean years of schooling	−0.34 (−0.83, 0.21)	−0.40 (−1.32, 0.29)
		p=0.207	p=0.275
GII	ln labour force participation	−0.17 (−0.55, 0.45)	2.10 (1.08, 3.01)
		p=0.580	p=0.000
GDI	ln gross national income per capita	−0.44 (−0.73, −0.12)	0.01 (−0.32, 0.32)
		p=0.009	p=0.994
GDI	ln life expectancy	−3.61 (−7.43, 0.07)	−0.26 (−4.18, 3.65)
		p=0.055	p=0.911

GDIGender Development IndexGIIGender Inequality Index

We repeated the analysis in table 1 with data sets that excluded (a) high-income countries (HICs), (b) Sub-Saharan African countries, (c) Latin America and the Caribbean (LAC), (d) Asian countries and (e) countries in Eastern and Central Europe and Central Asia (online supplemental appendix tables 2a–e). One variable that showed varying results in the regional jackknife estimates was the sex ratio in life expectancy: the models without HICs showed much stronger associations between this component of gender inequality and all reproductive outcomes, suggesting that these relationships are weaker or do not hold in HICs. In addition, the direct correlations between better gender ratios in educational attainment and the proportion of unintended pregnancies ending in abortion fell away in the models without sub-Saharan Africa. Otherwise, these results suggest our findings are robust to the inclusion or exclusion of any individual regions in the analytic data, and not driven by any one region.

We conducted cross-sectional analyses for the six 5-year time periods from 1990 to 2019. The relationship between disparities in educational attainment and unintended pregnancy, controlling for GDPPC, held in all periods. In models additionally controlling for other indicators of gender inequality, the associations between disparities in educational attainment and unintended pregnancy held for almost every 5-year period from 1990 to 2019 (online supplemental appendix tables 3a–e). Whereas the strength of the associations of unintended pregnancy with gender disparities in life expectancy and parliamentary representation varied across time periods, the relationship of disparities in educational attainment with unintended pregnancy appeared to get consistently stronger over time.

When we replaced the CUPR with the UPR as the dependent variable, the strengths of the associations were substantially attenuated (table 1). For example, the association of unintended pregnancy with the GII without its health component net of GDPPC in 2015–2019 decreased by a third, from 0.14 (0.03, 0.25) to 0.09 (−0.02, 0.18), and associations with educational attainment, GDI and GGI decreased by about half.

## Conclusions and discussion

We found that gender inequality, and particularly gender disparities in educational attainment, are directly correlated with the incidence of unintended pregnancy and unplanned births among women wanting to avoid pregnancy.

Our findings make a number of contributions to the evidence on the relationship between gender inequality and reproductive outcomes. First, we use a refined measure of unintended pregnancy, the CUPR, which essentially allows us to control for trends and differentials in the proportion of women who wish to avoid pregnancy across settings. Second, we are able to additionally examine correlations of gender inequality with the two main outcomes of unintended pregnancy: unplanned birth and abortion. Third, we are able to simultaneously examine different components, or manifestations, of gender inequality. Finally, our analyses cover a broad geographic scope, namely 132 countries across all of the major world regions over a 30-year time period.

We found that the CUPR, a measure of the incidence of unintended pregnancy among women who want to avoid pregnancy, paints a clearer picture of the relationship between gender inequality and unintended pregnancy than does the more conventional UPR, which measures the incidence of unintended pregnancy among all women of reproductive age. This can be expected because the CUPR accounts for the possibility that the proportion of women wanting to avoid pregnancy will increase as gender gaps diminish—a phenomenon that would manifest in lower CUPRs but not in lower UPRs.

A recent cross-national assessment of induced abortion and gender inequality in Europe found that gender inequality was positively associated with the rate of abortion per 1000 women of reproductive age.^23^ In our examination of gender equality and the proportion of unintended pregnancies that end in abortion, we find mixed results. Since, all else being equal, abortion rates will be higher where unintended pregnancy rates are higher, associations between abortion rates and gender inequality may reflect the relationship between unintended pregnancy and gender inequality.

Prior analyses have shown that schooling for females is inversely associated with women’s ideal family size^24^ and fertility.^25^ We add to this evidence by demonstrating that gender disparities in schooling and educational attainment for females net of schooling for males, are inversely correlated with women’s ability to avoid unintended pregnancies and unplanned births. The correlation between educational disparities and unintended pregnancy remained strong even after controlling for disparities in labour force participation and income, suggesting that educational attainment operates through channels aside from women’s participation in the workforce and income. As prior research suggests, the pathways through which schooling decreases the incidence of unintended pregnancy can vary across settings,^24^ and could include education’s effects on women’s participation in decision-making and their knowledge about their contraceptive options.

The finding that female-specific indicators of educational attainment, as well as life expectancy and GNIPC, were more strongly associated with CUPRs than were male-specific indicators is consistent with prior research^26^ and underscores the critical importance of including women in development strategies.

Though gender inequalities in educational attainment were the component measures most consistently associated with unintended pregnancy in multivariable analyses, this does not mean that other aspects of gender inequality are unrelated to unintended pregnancy. We found significant associations between unintended pregnancy and gender inequalities in life expectancy and parliamentary representation during some time periods. In addition, female GNI per capita became strongly associated with unintended pregnancy in the model controlling for male GNI per capita and overall GDP per capita. Null results could also reflect limitations in the data underlying the indicators. For example, gender ratios in GNI may not capture the economic activities of males and females equally well, especially in regions with large informal economies where women’s contributions might be under-reported or undervalued. While there was some variability in the strength of the associations of the component measures depending on regression model specification and time period, in all our analyses, regardless of model specification or time period, the composite measures were significantly associated with the CUPR.

Other studies have explored the relationship between gender inequality or women’s empowerment (using various measures of empowerment) and fertility or unwanted fertility.^27 28^ Most of these studies have used surveys of women, and most used outcomes aside from unintended pregnancy, such as unplanned births (which omit abortions), overall births or even desired family size. Our analysis builds on the conclusions of much of the existing literature by pointing to a direct correlation between gender inequality and unintended pregnancy across countries over a 30-year period.

The sharp decline in the CUPR in Sub-Saharan Africa speaks to the success of programmes aimed at providing individuals and couples with the opportunities and resources they need to avoid pregnancy. It indicates that programmes have been more impactful in helping women and couples achieve their fertility aspirations than we might otherwise understand. These impacts have been masked by simultaneous increases in the demand for these services—most likely arising from social and economic development and their effects on women’s interest in controlling the size of their families and the timing of their births.

However, there is still a long way to go in both understanding and addressing unintended pregnancy and reproductive agency more broadly.

For one, the correlations shown here are based on ecological analyses. As such, they do not necessarily demonstrate a causal link between gender equality and its components on the one hand and unintended pregnancy on the other. Disentangling causal pathways that can lead to solutions requires extensive research using various methods. In addition, these analyses examined inequities at the country level, not within households. Inequities between couples (not measured here) could influence fertility outcomes through different pathways than country-level inequities.

Second, the numerator of the unintended pregnancy rate is subject to biases and measurement errors, as others have described in the existing literature.^10 13 15 16 29 30^ The estimates draw heavily from women’s responses to survey questions. These questions may not fully capture women’s ambivalence about pregnancies or the fact that ideas about wantedness could be culture-specific and vary across settings. To help account for the uncertainty this creates in global comparative analysis, the Bayesian model used to estimate the incidence of unintended pregnancy included terms for sampling *and* non-sampling error in the underlying data, and we sampled from this model’s posterior during the bootstrap procedure for our regression analyses.^10 11 16^

Third, while the CUPR uncovers previously masked progress, another important lesson is that policymakers and practitioners should not rely on only one measure of this important reproductive health outcome. For example, the UPR provides a snapshot of the population prevalence of unintended pregnancy and can help guide investments in relevant programmes.

Finally, country and regional measures do not expose differentials between population subgroups, which can be defined by age, ethnicity, socioeconomic status and other delineations. Disaggregated measures are critical to helping us identify and reach those persistently left behind. In some countries, this will require surveys with large samples of women, alongside data collection on the characteristics of respondents—the standard information like their age, wealth, education and place of residence, but also on whether they belong to marginalised ethnic groups, live with disabilities or have other characteristics that increase peoples’ risk of marginalisation.

Some unintended pregnancies follow from contraceptive failure, whether method failure or user failure;^6^ some unintended pregnancies are the result of women’s freely made decisions not to use a contraceptive method, and some are a consequence of contraceptive non-use for reasons such as limited access to quality services or limited reproductive autonomy.^31^ The strong relationship between gender inequality and the incidence of unintended pregnancy among women wanting to avoid pregnancy suggests that reproductive agency, which stems from factors such as access to education, economic empowerment and autonomy in decision-making, could significantly influence women’s ability to act on their reproductive preferences. Additional research on the factors that inhibit people’s ability to implement their reproductive preferences can help policymakers and practitioners refine strategies aimed at ensuring that every woman has the opportunity to make informed choices about her reproductive health.

## supplementary material

10.1136/bmjgh-2024-016573 online supplemental file 1

10.1136/bmjgh-2024-016573 online supplemental file 2

10.1136/bmjgh-2024-016573 online supplemental file 3

10.1136/bmjgh-2024-016573 online supplemental appendix 1

## Data Availability

Data are available upon request.
