# Supplementary material for: Unintended pregnancy and gender inequality worldwide: an ecological analysis
Source: BMJ Glob Health. 2025 Mar 31;10(3):e016573. doi: 10.1136/bmjgh-2024-016573 (PMC11962802; doi:10.1136/bmjgh-2024-016573)
Supplement: online supplemental file 1 [file bmjgh-10-3-s001.pdf]

Appendix Table 1. Countries in analysis, 2015-2019

Country	Conditional unintended pregnancy rate [a]	Unintended pregnancy rate [a]	Gender Inequality Index [b]	Gender Development Index [b]	Gender Gap Index [c]
Angola	0.291	0.124	0.538	0.900	0.640
Albania	0.039	0.016	0.170	0.991	0.728
Argentina	0.105	0.070	0.328	0.993	0.732
Armenia	0.117	0.054	0.261	0.990	0.677
Australia	0.057	0.038	0.096	0.966	0.731
Azerbaijan	0.216	0.095	0.311	0.951	0.676
Burundi	0.279	0.100	0.510	0.930	0.755
Belgium	0.033	0.021	0.057	0.973	0.739
Benin	0.215	0.088	0.615	0.871	0.652
Burkina Faso	0.163	0.076	0.600	0.893	0.646
Bangladesh	0.095	0.059	0.534	0.902	0.719
Bulgaria	0.041	0.029	0.208	0.993	0.756
Bosnia and Herzegovina	0.044	0.021	0.162	0.922	0.702
Belarus	0.029	0.019	0.111	1.010	0.744
Belize	0.131	0.074	0.414	0.980	0.692
Bolivia	0.185	0.108	0.424	0.950	0.758
Brazil	0.098	0.069	0.421	0.990	0.684
Barbados	0.129	0.080	0.289	1.033	0.750
Bhutan	0.107	0.049	0.473	0.933	0.638
Botswana	0.156	0.099	0.468	0.985	0.720
Central African Republic	0.237	0.094	0.691	0.805	—
Canada	0.041	0.032	0.083	0.987	0.769
Switzerland	0.027	0.021	0.034	0.967	0.755
Chile	0.107	0.072	0.243	0.965	0.704
China	0.091	0.067	0.217	0.981	0.674
Cameroon	0.204	0.087	0.578	0.876	0.689
Congo	0.196	0.111	0.566	0.935	—
Colombia	0.093	0.063	0.427	0.991	0.731
Cabo Verde	0.183	0.107	0.385	0.982	0.686
Costa Rica	0.083	0.052	0.307	0.980	0.727
Cuba	0.093	0.072	0.310	0.958	0.745
Germany	0.033	0.022	0.087	0.971	0.778
Denmark	0.044	0.031	0.027	0.981	0.776
Dominican Republic	0.130	0.085	0.454	1.006	0.697
Ecuador	0.128	0.082	0.376	0.965	0.724
Spain	0.030	0.020	0.068	0.984	0.746
Estonia	0.060	0.038	0.106	1.024	0.731
Ethiopia	0.193	0.079	0.525	0.903	0.656
Finland	0.040	0.033	0.042	0.989	0.823
Fiji	0.151	0.070	0.340	0.935	0.638
France	0.042	0.029	0.073	0.989	0.778
Gabon	0.209	0.116	0.549	0.907	—
United Kingdom	0.047	0.036	0.117	0.972	0.770
Georgia	0.217	0.097	0.309	0.983	0.679
Ghana	0.231	0.105	0.538	0.857	0.695
Guinea	0.237	0.072	0.614	0.845	0.659
Gambia	0.203	0.054	0.611	0.904	0.649
Guinea-Bissau	0.242	0.105	0.630	0.870	—

Country	Conditional unintended pregnancy rate [a]	Unintended pregnancy rate [a]	Gender Inequality Index [b]	Gender Development Index [b]	Gender Gap Index [c]
Greece	0.057	0.034	0.129	0.970	0.692
Guatemala	0.127	0.063	0.520	0.929	0.667
Guyana	0.159	0.078	0.465	0.971	—
Honduras	0.119	0.070	0.424	0.972	0.711
Croatia	0.060	0.035	0.130	0.985	0.711
Haiti	0.218	0.110	0.637	0.902	—
Hungary	0.052	0.028	0.248	0.980	0.670
Indonesia	0.080	0.042	0.455	0.933	0.691
India	0.123	0.063	0.510	0.851	0.669
Ireland	0.048	0.034	0.086	0.976	0.794
Italy	0.044	0.029	0.070	0.968	0.692
Jamaica	0.178	0.089	0.372	0.991	0.717
Japan	0.037	0.022	0.093	0.970	0.657
Kazakhstan	0.113	0.060	0.177	0.992	0.713
Kenya	0.196	0.113	0.515	0.936	0.694
Kyrgyzstan	0.102	0.041	0.380	0.959	0.691
Cambodia	0.152	0.073	0.470	0.919	0.676
South Korea	0.059	0.036	0.080	0.938	0.650
Laos	0.118	0.054	0.471	0.959	0.703
Liberia	0.215	0.110	0.655	0.857	0.669
Saint Lucia	0.121	0.071	0.394	1.017	—
Sri Lanka	0.083	0.041	0.372	0.962	0.669
Lesotho	0.163	0.102	0.560	0.989	0.695
Lithuania	0.050	0.027	0.133	1.020	0.742
Latvia	0.051	0.034	0.204	1.030	0.756
Moldova	0.045	0.027	0.242	1.015	0.740
Madagascar	0.181	0.096	0.554	0.951	0.692
Maldives	0.175	0.064	0.346	0.934	0.669
Mexico	0.095	0.061	0.336	0.959	0.692
Mali	0.190	0.069	0.676	0.849	0.583
Myanmar	0.091	0.037	0.513	0.955	0.691
Montenegro	0.026	0.011	0.124	0.972	0.693
Mongolia	0.059	0.032	0.326	1.025	0.713
Mozambique	0.215	0.090	0.544	0.912	0.741
Mauritania	0.239	0.069	0.625	0.883	0.614
Malawi	0.190	0.115	0.576	0.962	0.672
Namibia	0.177	0.109	0.449	1.003	0.777
Niger	0.169	0.050	0.637	0.826	—
Nigeria	0.226	0.069	0.670	0.861	0.641
Nicaragua	0.100	0.057	0.431	0.958	0.814
Netherlands	0.026	0.018	0.029	0.964	0.737
Norway	0.048	0.034	0.023	0.989	0.830
Nepal	0.103	0.060	0.469	0.931	0.664
New Zealand	0.059	0.041	0.112	0.966	0.791
Pakistan	0.206	0.072	0.543	0.754	0.546
Panama	0.123	0.075	0.417	1.007	0.722
Peru	0.156	0.091	0.404	0.951	0.719
Philippines	0.160	0.072	0.423	0.981	0.790
Poland	0.051	0.030	0.125	1.006	0.728
Portugal	0.031	0.021	0.077	0.987	0.734

Country	Conditional unintended pregnancy rate [a]	Unintended pregnancy rate [a]	Gender Inequality Index [b]	Gender Development Index [b]	Gender Gap Index [c]
Paraguay	0.108	0.070	0.454	0.971	0.678
Romania	0.080	0.048	0.283	0.982	0.708
Russia	0.122	0.067	0.219	1.005	0.696
Rwanda	0.224	0.098	0.393	0.921	0.822
Senegal	0.173	0.061	0.531	0.880	0.684
Singapore	0.043	0.020	0.048	0.989	0.702
Sierra Leone	0.204	0.092	0.640	0.887	—
El Salvador	0.101	0.059	0.372	0.982	0.705
Serbia	0.037	0.022	0.138	0.976	0.727
South Sudan	0.222	0.056	0.597	0.851	—
Sao Tome and Principe	0.238	0.135	0.517	0.910	—
Suriname	0.144	0.068	0.440	1.002	0.689
Slovakia	0.047	0.028	0.193	0.991	0.694
Slovenia	0.057	0.034	0.054	0.994	0.805
Sweden	0.053	0.036	0.035	0.984	0.816
Chad	0.249	0.061	0.699	0.764	0.575
Togo	0.223	0.102	0.588	0.840	—
Thailand	0.069	0.041	0.419	0.995	0.694
Tajikistan	0.104	0.040	0.280	0.904	0.678
Turkmenistan	0.052	0.024	0.184	0.955	—
Timor-Leste	0.167	0.053	0.394	0.918	0.628
Tonga	0.161	0.053	0.363	0.968	—
Trinidad and Tobago	0.162	0.086	0.348	0.986	—
Tanzania	0.210	0.105	0.563	0.939	0.700
Uganda	0.281	0.145	0.534	0.888	0.721
Ukraine	0.048	0.029	0.260	1.014	0.705
Uruguay	0.067	0.042	0.269	1.018	0.710
United States	0.050	0.035	0.209	0.990	0.718
Uzbekistan	0.056	0.030	0.278	0.944	—
Venezuela	0.110	0.069	0.481	1.009	0.706
Samoa	0.166	0.071	0.407	0.984	—
South Africa	0.140	0.085	0.407	0.979	0.756
Zambia	0.241	0.123	0.540	0.993	—
Zimbabwe	0.131	0.074	0.532	0.952	0.717

[a] Bearak et al, 2022
[b] United Nations Development Programme Human Development Report
[c] World Economic Forum Global Gender Gap Report
